# Food-Derived Phytochemicals: Multicultural Approaches to Oxidative Stress and Immune Response

**DOI:** 10.3390/ijms26157316

**Published:** 2025-07-29

**Authors:** Eiger Gliozheni, Yusuf Salem, Eric Cho, Samuel Wahlstrom, Dane Olbrich, Brandon Shams, Michael Alexander, Hirohito Ichii

**Affiliations:** Department of Surgery, University of California Irvine, Orange, CA 92868, USA; egliozhe@uci.edu (E.G.); ymsalem@hs.uci.edu (Y.S.); ejcho5@hs.uci.edu (E.C.); swahlstr@uci.edu (S.W.); dolbrich@uci.edu (D.O.); bdshams@uci.edu (B.S.); michaela@hs.uci.edu (M.A.)

**Keywords:** antioxidant, Nrf2/Keap1 pathway, Th1/Th2 ratio, inflammation, oxidative stress

## Abstract

This review will focus on how ethnic consumption of foods such as shiitake, ginseng, turmeric, black seeds, berries, rosemary, moringa and holy basil can help act as antioxidants and immune modulators in fighting many diseases. We will investigate how these foods act on pathways like Nrf2/Keap1 to increase endogenous antioxidant capacity and help in reducing ROS production, based on publications found in PubMed between 1994 and 2024. In addition, we will show how these plants can cause immune system shifts by changing the makeup of the ratio of Th1/Th2 cells, reduce inflammation, and have antiangiogenic effects on cancer. This review will also show how plants can alter the gut microbiota and lead to a further decrease in oxidative stress. Overall, it will show how plants and their metabolites can potentially create a path forward for creating novel therapeutic approaches and help lead to an improved redox balance, support immune function, and enhance long-term health outcomes.

## 1. Introduction

Over the past few decades, researchers have conducted a significant number of studies on oxidative stress and its contribution to chronic diseases, such as cancer, diabetes, cardiovascular disease, and neurodegeneration. Oxidative stress is a pathophysiological state that occurs when reactive oxygen species (ROS) production exceeds the body’s endogenous antioxidant defense mechanisms and causes cellular damage and consequent systemic inflammation. In addition, it has been postulated that certain dietary and ethnobotanical treatments, defined as the traditional use of plants as medicine and food, from traditional societies can help restore redox balance and, in some cases, enhance immune capacity.

This review will examine the antioxidative and immunotherapeutic effects of the following seven medicinal plants: shiitake mushroom, ginseng, turmeric, black seed, berries, moringa, and holy basil. These plants were chosen on the premise they have historical significance, documentable use in traditional medicine across a diverse range of cultures, and these anecdotal claims have been supported in recent scientific research. The primary mediator of the potential therapeutic utility of these plants is the modulation of the Keap1/Nrf2 (Kelch ECH-associating protein 1/nuclear factor erythroid 2-related factor 2) axis, and subsequently, the restoration of cellular antioxidative defense capacity. Additionally, these phytochemicals also modulate various aspects of immune homeostasis, including Th1/Th2 modulation, the inhibition of pro-inflammatory cytokines, and the stimulation of beneficial gut microbiota, as summarized in Table 1. Collectively, these findings can indicate significant health benefits with important implications for disease prevention and integrative therapy.

In essence, we provide a review of the current literature on these plant-based compounds with regard to their antioxidative/immunoregulatory action, with the aim to understand the mechanistic potential for future research.

## 2. Lentinan

*Lentinus edodes* (shiitake mushroom) has been shown to reduce oxidative stress in a number of diseases, including cancer [1]. One targeted therapy for cancer is via immunotherapy using bioactive β-glucans [2]. A higher Th1/Th2 ratio has been shown to improve outcomes [3,4]. Th2 cells also release IL-4 and IL-10, anti-inflammatory interleukins which suppress the immune system’s ability to fight cancer, since inflammation allows for the signaling of macrophages and CD8+ T cells to fight infections or the causes of cellular damage. Therefore, by having a higher Th2 ratio, this leads to the opposite effect than that which is required to fight cancerous cells, because this release of anti-inflammatory interleukins, specifically Il-4, leads to decreased development and suppression of the maturation of Th1 cells.

Due to Th1 being part of the cell-mediated immune response (CMI), it is crucial in fighting many viral and bacterial infections, as well as cancer. Th1 cells release IL-2, which plays an essential and recursive role in causing the production of other cytokines that are essential to fight cancer. IL-2 causes an increased release of IFN-γ and TNF-α and the development of CD8+ T-cells, and both are necessary to attack cancer cells [5]. By increasing IFN-γ production, Th1 activation increases the presentation of MHC I receptor cell surfaces, which makes it easier for killer T-cells to recognize cancer cells for destruction. Additionally, Th1 activation produces TNF-α, which promotes the apoptosis of cancerous cells by binding to TNFR1 and TNFR2, helping to create a microenvironment which invites the CMI (CTL and NK cells) to help eliminate cancerous cells [6]. TNF-α also plays an opposite role in Th2 cells by eliminating blood supply to these cancerous cells, essentially starving them to death [7].

### 2.1. Lentinan Mechanism of Action

The importance of bioactive β-glucans, and specifically β-(1→3)-glucan with β-(1→6) branching, has been shown to modulate the immune response towards favorable high Th1/Th2 ratios, which are necessary for the body to be able to effectively fight cancer. In the case of lentinan, it does this by activating TLRs and increasing the signal strength of APCs, which makes it easier for Th1 cells looking to kill cancerous cells to be able to identify and eliminate these types of cells [5]. This has been shown to be the case in individuals with lung adenocarcinoma, whose ratio of Th1 cells increases significantly in the MPE (malignant pleural effusion) caused by lung cancer. This changes the cellular environment from one that is considered cold (immune system not actively looking for cancerous cells) to a hot one, where due to the increase in Th1 cells and the cytokine environment, they lead to the cancer being aggressively attacked [8]. This effect has also been seen in patients with NSCLC, who tend to have irregular T cell profiles and cytokine production, which generally leads to them having a low Th1/Th2 ratio. When given lentinan as a supplemental treatment, compared to the single-chemotherapy group, the amount of NK and CD8+ T cells in the lentinan-supplemented group almost doubled, reaching 15.7  ±  3.1% vs. 8.6  ±  1.4%, and the essential CMI cytokines (IFN-γ and TNF-α) saw a significant increase, with *p* < 0.05 [9].

**Figure 1:**
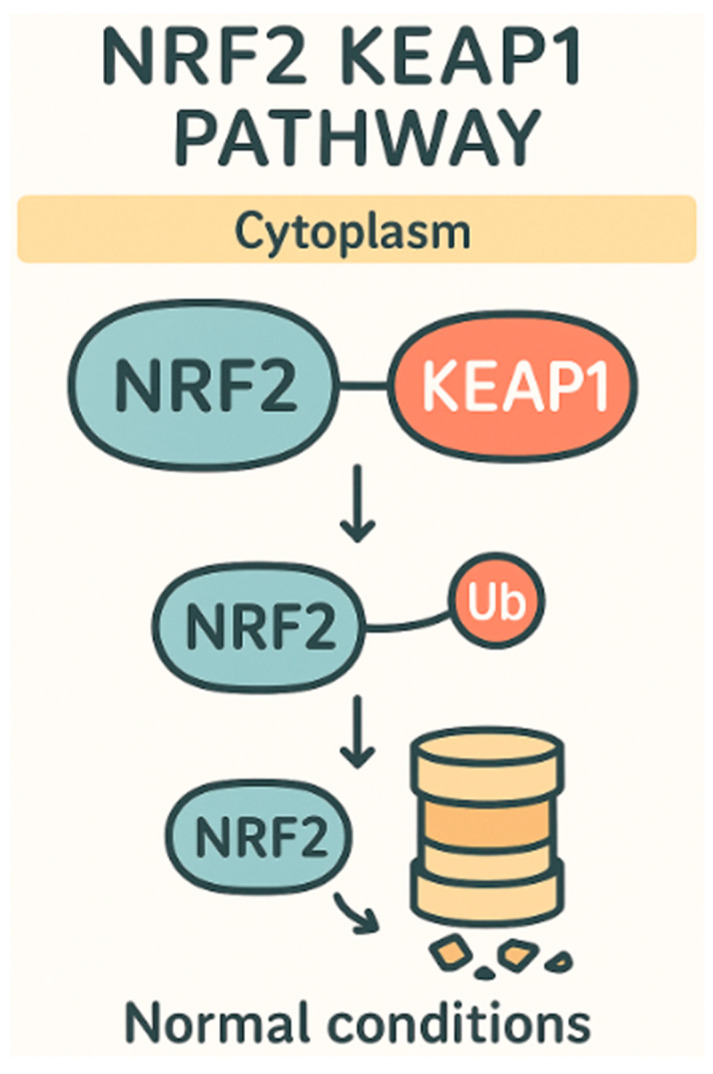
Nrf2-Keap1 pathway under normal conditions.

**Figure 2:**
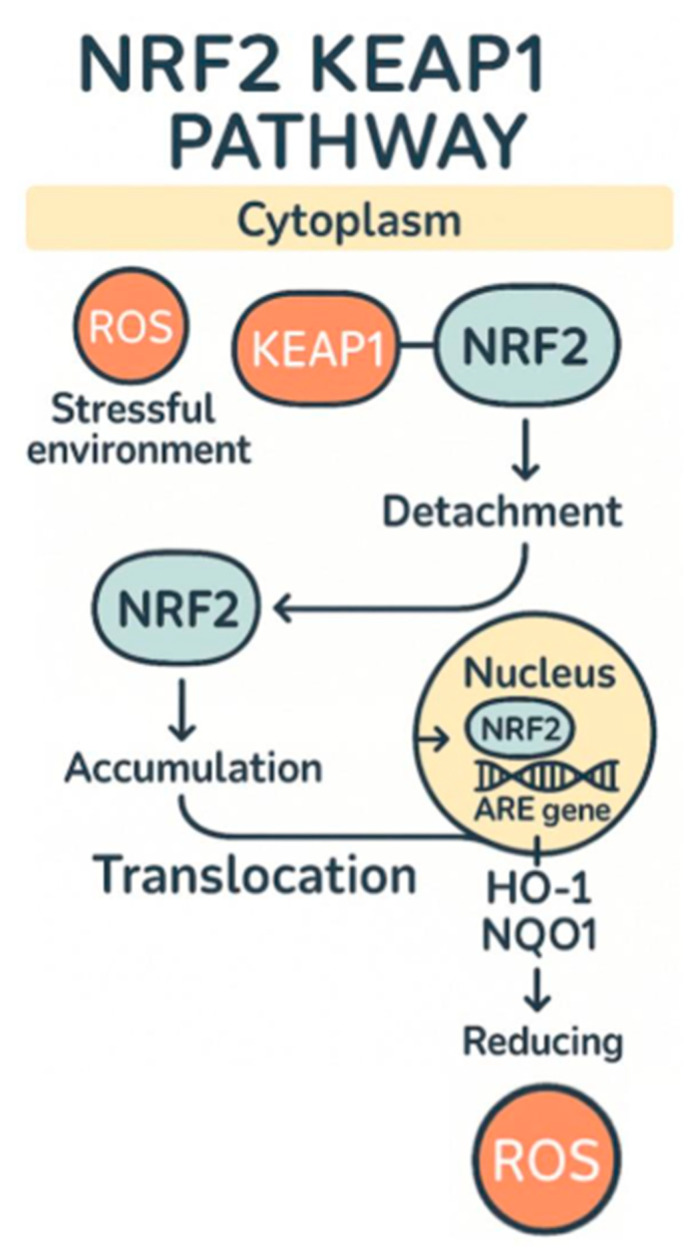
Nrf2-Keap1 pathway under oxidative stress.

Another area in which lentinan has been shown to effectively reduce oxidative stress is in individuals suffering from IBD (irritable bowel disease), and it is able to do this through a variety of methods. One of the ways in which it reduces oxidative stress caused by IBD is by decreasing expression of IL-13 and CD30L in mice that had dextran sodium sulfate (DSS)-induced colitis [6]. This resulted in maintenance of colon lengths compared to the model mouse and in a significant decrease in colon length in the untreated group who had DSS-induced colitis. In addition to this, it was observed that the microscopic damage which the DSS induced was significantly higher than the lentinan-treated group and control group *p* < 0.05. The damage was 300% higher than the lentinan group [10]. In cases in which colitis is caused by CAC (cancer-associated colitis), it was also observed that through a decreased expression of TLR4 and NF-κB, lentinan was able to reduce the amount of tumors present by 270% compared to the CAC group [10]. It is also believed that another one of the major causes of IBD is the imbalance of gut microbiota which is caused by dysbiosis, and lentinan has also been shown to make changes to the gut microbial environment, which can lessen IBD’s symptoms [11]. Lentinan acts as a prebiotic, and when damage is induced by dysbiosis, lentinan is able to reduce its effects because of the decreased NF-κB expression, increased number of actinobacteria, and decrease in the harmful proteobacteria and epsilon bacteremia, which has been linked to causing colitis due to being increased in numbers of affected IBD individuals [12,13]. The final method in which lentinan has been shown to reduce oxidative stress caused by IBD is via the Nrf2 pathway (Figure 1). It does this by reducing OX40/IL-17A signals, which increases inflammation and oxidative stress by inciting a cytokine storm of sorts using NF-κB as a precursor to the storm [14]. As mentioned, the reason it is able to enact this change is because of Nrf2 pathways, and specifically activation of nuclear factor erythroid 2 (NFE2), allowing for the translation of the NQO1 protein, which reduces quinones to hydroquinones and thereby halts the production of ROS in the cell [15,16] (Figure 2).

### 2.2. Lentinan Administration and Contradcitions

When prescribing any kind of treatment, it is always important to consider the method of administration and if these methods have the potential to cause unforeseen effects. Lentinan has proven to be effective via two different routes of administration. The first of which includes IV administration at a dosage of 2–10 mg per week over a 30 min period to minimize side effects such as anaphylactoid reaction, rigor, fever, pain, and chills [17]. The other method by which lentinan may be administered is via capsule at 8 g/day. This method showed no side effects; however, the effectiveness of the method was not as pronounced as the IV method when it came to causing significant patient outcomes [18]. Furthermore, when using lentinan, one must also take into consideration contradictions against different groups. One such group is individuals with mushroom allergies to begin with, as this can lead to a type 1 hypersensitivity reaction, which leads to anaphylaxis and could lead to death if appropriate medical interventions do not take place [19]. Another group who should avoid lentinan or require careful monitoring would be those whose drugs are metabolized by CYP450 enzymes (CYP3A4, CYP2D6, CYP2C19, CYP2C9, and CYP1A2) [20]. This includes those on anticoagulants, antihypertensives, and antiarrhythmics. Specifically, those at highest risk would be those on warfarin, a vitamin K antagonist that normally on its own would require prothrombin (PT) or international normalized ratio (INR) monitoring. But given that at low doses, lentinan increased CYP450 activity, and at high doses, it inhibited CYP450, it would be better to simply avoid lentinan for these patients, as the importance of maintaining a non-hypercoagulable state is pertinent in stopping one of Virchow’s triad [21], or those on β-blockers, such as metoprolol for antihypertensive or antiarrhythmic reasons. Lentinan should also be avoided as it showed a significant increase in CYP2D6 activity, which is the enzyme that metabolizes metoprolol [20]. This would obviously have the unintended effect of requiring a higher consumption of metoprolol and a less effective protective period for patients. Therefore, when considering whether lentinan should be used in conjunction with these other essential drugs, it would be more pertinent for the patient to maintain their usage of these therapies and discontinue their use of lentinan for therapeutic effects.

## 3. Panax Ginseng

*Panax ginseng*, a plant native to China and Korea, has been shown to alleviate many oxidative stress-induced diseases. One of these diseases is diabetic nephropathy, which is caused by AGEs (advanced glycation end-products) due to the result of a high sugar concentration being constant in the blood and attaching to proteins and fats [22]. This leads to the activation of several different pathways which produce ROS and increases the cellular damage inflicted on the kidney [23]. In addition to the cellular-level damage, it also affects kidney functions. AGE damages the nephrons ability to filter out proteins from the blood, which causes proteinuria, and furthermore leads to hypertension and decreased GFR (glomerular filtration rate), which is essential in fighting the buildup of AGEs because a higher GFR is necessary to rid the kidney of harmful proteins, which can create the environment necessary for AGEs [24].

### 3.1. Ginseng Mechanism of Action

Prior to using ginseng as a nutraceutical, it is steamed to reach the temperature of 100 °C, colloquially known as red ginseng [25]. This increases its bioavailability and efficacy by activating the saponins Rg1 and Re, which are the secondary metabolites that have been shown to counter diabetic nephropathy [26]. In patients with spontaneous hypertension, their blood vessels are forced to expand and contract many times. These patients had high amounts of secondary kidney damage, and as a result, this led to decreased GFR rates. GFR decrease has been shown to lead to the compounding of the effects of damaged kidneys and increased AGEs production. Rg1 showed incredible outcomes in fighting off the damage caused by spontaneous hypertension. It was able to structurally change the inner aorta to have a smaller diameter and decreased the resistance of the inner artery walls by making them thinner *p* < 0.001 [27]. This means that the two major damaging pathways of spontaneous hypertension can be reversed by the secondary metabolite Rg1 by recovering the flexibility of the arterial vessel.

The next ginseng metabolite, Re, has been shown to have a positive effect on a streptozotocin (STZ)-induced diabetes model. In a mouse model treated with low-dose 20 mg/kg STZ, which induces beta cell death, Re treatment resulted in lower blood glucose and total cholesterol levels after 8 weeks of Re supplementation, and increased glutathione levels in the kidney, compared to control diabetic mice [28]. In this study, *Panax ginseng* is prepared via using the extraction method, where 100 g of the flower bud, main root, and fibrous roots are placed in distilled water for 600 mL for 1 h, 800 mL for 1 h and 1000 mL for 2 h, respectively. This was then filtered and combined for freeze-drying at −20 °C until use. The ginsenoside profile of this method yields a higher concentration of Rb1 secondary metabolite [29], which is not the primary metabolite produced via the red ginseng preparation method. However, it does share the antioxidative protective effects of *Panax ginseng* we have discussed thus far. In a glycerol-induced acute renal failure (ARF) study performed on mice, it was found that in mice that received Rb1 (ARF + GS) for 48 h, creatinine (CRE) and blood urea nitrogen (BUN) were significantly reduced compared to the ARF + NS group, and showed comparable BUN and CRE to the control group (NS + NS), which did not receive glycerol-induced ARF treatment. This antioxidative effect of Rb1 was further supported by the fact that the measurement of MDA and GSH levels in rats that received Rb1 metabolite for 48 h showed significantly less lipid peroxidation, as measured by their reduced MDA levels compared to ARF + NS group. It also showed a restored GSH level compared to the ARF + NS group (*p* < 0.05). Finally, this was also supported by the histological findings where the ARF + NS group showed membrane blebbing and tubular necrosis consistent with irreversible cell damage versus the ginsenoside-treated group, which maintained normal cell morphology [30]. This shows the wide variety of antioxidative effects that the Rb1 metabolite can have on several different cellular process which lead to cell death. In another study where an intestinal ischemia-induced reperfusion injury study was performed and the Rb1 metabolite was used to test its antioxidative effects, when measuring for BUN, it was shown that the intestinal-induced reperfusion group (IIR) compared to (IIR + GS) had significantly higher concentrations of BUN compared to those treated with the Rb1 group (21.55 ± 1.50 vs. 12.91 ± 1.41 mmol/L). Additionally, when comparing CRE levels, significantly reduced CRE levels were shown in the Rb1-treated group compared to the IIR group (13.14 ± 1.22 vs. 20.65 ± 1.53 μmol/L). Furthermore, when comparing MDA levels, they also found that the Rb1-treated group had significantly lower levels than the IIR group (*p* < 0.05). Finally, when performing immunohistology chemistry, they found that compared to the control and IIR group, the Rb1-treated group had activated the Nrf2/KEAP pathway significantly more, and produced significantly more HO-1 than either of the two groups as was visually evidenced by the significantly more brown color due to antibody-binding HO-1 [31]. The final metabolite found within *Panax ginseng* that requires a unique processing method is Rd. It used the ginseng berries and ginseng white root, cleaned them using tap water, and then vacuum-sealed them. The berries and roots were then twice subjected to ultra-high pressure (UHP) using water at 550 MPa for 1 min. Samples were then steamed at 95 °C for 3 h and dried at 53 °C for 70 h to produce a red ginseng high in Rd metabolite concentration. This sample was then pulverized and stored at −20 °C until use [32]. Rd’s effects have been studied in several renal injury models, and throughout them all, it has shown highly nephroprotective effects. In one study where cephaloridine-induced renal disorder was tested, Rd-treated mice showed significantly reduced BUN levels from 32.1 ± 2.3 to 25.0 ± 2.3 mg/dL and significantly reduced CRE levels from 0.81 ± 0.05 to 0.46 mg/dL. The Rd metabolites’ highly antioxidative effects were further backed up by its MDA levels post-Rd-treatment, which showed significant decreases from 64.9 ± 3.1 to 50.9 ± 3.0 nmol/g. This showed Rd’s antioxidative effects in reducing lipid peroxidation. In addition to this, the histological findings supported this finding by showing reduced necrotic lesions and tubuloepithelial damage compared to the control [33,34]. Rd’s antioxidative effects were also observed in another IIR study, which showed significant decreases in the IIR + GS in BUN from 32.1 ± 2.1 to 25.0 ± 2.3 mg/dL. This trend was also observed in CRE, where levels decreased from 0.81 ± 0.05 to 0.46 ± 0.02 mg/dL compared to the IIR group. In continuation with this trend, it was also observed that MDA showed a significant decrease from 64.9 ± 3.1 to 50.9 ± 3.0 nmol/g [35]. Given the extensive research performed on *Panax ginseng* and its metabolites, it is apparent that its antioxidative properties are clinically significant, and could be used in the treatment of chronic diseases that cause large amounts of ROS production in the body.

### 3.2. Panax Ginseng Administration and Contraindications

As mentioned before, choosing the correct administration method and looking out for patient contradictions are essential in medical care, and *Panax ginseng* is no exception to this rule. Starting with IV administration of *Panax ginseng*, there seem to be gaps in this area related to safety data. However, in a 2019 review of *Panax ginseng*, it was found that there were no adverse reactions upon an administration of 1.0–2.0 mL per session 1–3 times a week. They also maintained that they lacked dose standardization in this study, and therefore cannot put much trust in the validity of this data [36]. The other administration method that has been studied significantly more is via oral administration, where 1 g/twice a day of *Panax ginseng* was used in combination with warfarin to study its effects on INR reduction. In this study, it showed a remarkable decrease in INR (−0.36 to −0.07) [37]. Given this significant effect, it seems necessary that we discuss the contradictions that it could have for potential patients, such as those on anticoagulants and those with known plant allergies. Continuing our previous discussion on the interaction between warfarin and *Panax ginseng*, it was shown to have had a significant effect on INR reduction, which is something that causes large concern, especially for those on warfarin whose INR needs to be carefully monitored. Specifically, it does this by activating CYP450 (CYP3A4 enzyme), which leads to a reduced circulation and activity of warfarin, which is essential to help maintain the non-hypercoagulable state. Therefore, in order to avoid requiring even higher levels of monitoring, it seems better to discontinue the use of *Panax ginseng* in combination with warfarin than run the risk of unforeseen complications [38]. Additionally, individuals who are allergic to *Panax ginseng* should avoid use due to the possibility of respiratory depression and hypotension possibilities, as this could lead to their direct death if not treated in a timely manner [39].

## 4. Turmeric

*Curcuma longa*, more commonly known as turmeric, is a plant native to the Indian subcontinent that has been used as a spice and medicinal compound in ancient medicine for thousands of years [40]. It has a wide range of reported health benefits, with reports of anticancer, anti-inflammatory, and antioxidative effects [41]. Turmeric has been widely reported for its positive effects on reducing oxidative damage in a wide range of diseases [42]. This section aims to elucidate turmeric’s capacity as an effective therapeutic compound through an antioxidative pathway.

### 4.1. Chemical, In Vitro, and In Vivo Antioxidative Characteristics

Curcumin is the active molecule of the greatest interest in turmeric, and it has been shown to act as an antioxidant in vitro. In a study conducted by Ak et al., it was demonstrated to act as an antioxidant via the free radical absorption and chelation of highly oxidized metals under various conditions in antioxidant assays [43]. There are several mechanisms by which curcumin can react with these ROS to undergo oxidation, stemming from the hydroxyl, methoxy, and carbonyl groups present in the compound, as outlined by Keramat et al. in their study examining curcumin’s antioxidant potency in vitro [44]. Each of these groups acts as an electron acceptor, allowing for a reduction in ROS or the absorption of a free radical [44]. This evidence shows the potential mechanism by which curcumin can act as a direct antioxidant in living systems [43,44].

With its direct ability to act as an antioxidant firmly established, curcumin has also been shown to upregulate the nuclear factor E2-related factor 2 (Nrf2) pathway, which is present in humans and is responsible for the increased expression of genes associated with antioxidant enzymes [45,46]. In one study conducted by Lin et al., when treating a mouse-derived macrophage cell line with varying levels of hydrogen peroxide, they found increased cell viability and lower levels of oxidative stress at concentrations of 5 μM and 10 μM [47]. They also found a significant increase in Nrf2 protein levels and increased activity levels of catalase, superoxide dismutase, and glutathione peroxidase, all of which are antioxidant enzymes regulated by the Nrf2 pathway [47].

More direct evidence of Nrf2 activation has been provided by Liu et al. in their study examining curcumin’s ability to suppress colorectal cancer cell metastasis [48]. Initially, they displayed curcumin’s ability to induce senescence and apoptosis in these cancer cells in vitro via activation of the Nrf2 pathway, which promote the transcription of the *miR-34a/b/c* genes that prevent cancer progression [48]. They then injected these cells into immunodeficient mice after being treated for 48 h with curcumin, finding that tumor suppression continues to prevent lung metastasis [48]. Interestingly, they found that the Nrf2 pathway was activated via ROS induction of curcumin, as when paired with an antioxidant, the tumor suppression was rendered ineffective [48]. While counterintuitive to curcumin’s well-documented antioxidative potential, there are a few explanations for this phenomenon, which will be elaborated further below.

Evidence also points to ROS’ absorption and induction of antioxidative enzymes in vivo. Al-Rubaei et al. conducted an in vivo experiment examining the capacity of curcumin to counter hydrogen peroxide-induced oxidative stress in rats [49]. Specifically, they found that rats given 200 mg/kg of curcumin orally following hydrogen peroxide consumption had reduced serum markers of hepatic tissue damage due to oxidative stress and increased total antioxidant capacity [49]. They also saw an increase in levels of glutathione after treating the rats with curcumin, giving indirect evidence of Nrf2 activation, as glutathione is an antioxidative enzyme linked to the pathway [49,50].

In the sphere of studies conducted on humans, Jakubczyk et al. conducted a meta-analysis of randomized clinical trials where curcumin was given to human participants to understand its effects on total antioxidant capacity and levels of malondialdehyde, a human marker for oxidative stress [51]. A total of four studies were examined, and pure curcumin was administered orally and found to have these effects [51]. The authors of the study concluded that curcumin could be positively shown as an antioxidant when ingested by humans, but they acknowledged that there is a need for more long-term studies due to the conflicting evidence from a wide range of studies [51].

Finally, there have been efforts to improve the bioavailability of curcumin via piperine co-supplementation, which has shown the most promise in therapeutic benefit in humans. Piperine, a naturally occurring compound derived from pepper, was used alongside curcumin to help combat the oxidative stress experienced by ischemic stroke patients in a study conducted by Boshagh et al. [52]. The patients given 500 mg and 50 mg of curcumin and piperine daily had a significant reduction in carotid intima–media thickness and an increase in total antioxidant capacity compared to the patients given a placebo [52]. Due to the positive correlation between carotid intima–media thickness and oxidative stress, this study provides compelling evidence of the antioxidative potential of curcumin in humans [52]. Lastly, Sharifi et al. administered 500 mg of curcumin and 5 mg of piperine daily to nonalcoholic fatty liver disease patients and found improved markers of liver function in the experimental group [53]. Although they noted no significant change in measurable liver steatosis, they noted that the increased liver function could be due to the antioxidant and anti-inflammatory properties of curcumin, and that a change in steatosis could potentially be found if the patients were followed-up for a longer period of time [53].

### 4.2. Conclusions on Curcumin

Overall, a vast body of evidence gives credence to curcumin’s antioxidative potential through its direct ROS, free radical sequestering, and Nrf2 pathway activation. However, controversy remains regarding conflicting evidence regarding its ability to generate ROS and its low bioavailability in the human body. As mentioned above, there are many instances of curcumin being shown to induce cancer-reducing properties through an increase in ROS levels [48,54]. Nevertheless, it is important to contextualize the fact that these were found to occur in in vitro studies where the duration of the experiments could have been a key factor in finding increased ROS levels. Liu et al. found increased in vitro ROS levels after exposing their colorectal cancer cells for 48 h, and Li et al. found a similar increase in ROS with their various colorectal cancer cell lines after 24-h in vitro treatment with curcumin [48,54]. Neither experiment examined any ROS markers when testing the compound in vivo and ex vivo, but it is likely that the ROS increase is localized to the cancer cells and is not significant systemically over a longer period, as hypothesized by Xu et al. [55]. This is further supported by the numerous in vivo and human trial studies mentioned previously that found a significant improvement in ROS biomarkers, all of which examined their subjects for several weeks.

Another interesting aspect of the ROS-dependent Nrf2 pathway activation found in Liu et al.’s study [48] is that it is the only study that provides a direct mechanistic explanation for Nrf2 activation via curcumin. Although the in vitro and human studies mentioned show a positive correlation for antioxidative effects, and in some cases indirect evidence of Nrf2 activation, they are only able to speculate on how curcumin induces the production of antioxidative enzymes. If the ROS experienced by the cells in vitro is also found in in vivo use, it seems that the low-grade ROS generation could potentially trigger the Nrf2 pathway systemically in vivo, thereby leading to a higher level of antioxidative enzymes and lower overall oxidative stress. This is feasible as a similar mechanism is seen in humans after exercising, where a temporary increase in ROS leads to a long-term reduction in oxidative stress through an increase in the production of antioxidative enzymes through the Nrf2 pathway [56]. Therefore, given the evidence of systemic reduced oxidative stress and the potential of a hermetic effect, it can be concluded that curcumin is a net antioxidant in in vivo settings.

Finally, with the significant improvement in bioavailability found with the co-supplementation with piperine, it can be said that there is a significant case for the oral use of curcumin and piperine in managing diseases that would benefit from curcumin’s antioxidative effects [57]. More studies should be conducted to examine the long-term effects of curcumin plus piperine supplementation to create guidelines for its therapeutic use, in addition to illuminating any benefits in its use for disease prevention.

## 5. Black Cumin

*Nigella sativa*, colloquially referred to as black seed or black cumin, is a plant originating in southwest Asia that has been used for centuries in Middle Eastern and southwest Asian cuisine and traditional medicine [58]. It has a wide range of purported medicinal uses for many conditions, including inflammation, hypertension, and diabetes [59]. Moreover, the presence of thymoquinone as its main active component suggests a potential antioxidative effect in humans, which will be explored in the following section [59].

### 5.1. Chemical, In Vitro, and In Vivo Antioxidative Characteristics

There is substantial evidence of thymoquinone’s antioxidative potential. Sakib et al. conducted a study that displayed thymoquinone and black seed oil’s ability to scavenge superoxide radicals through an electrochemical experiment [60]. Through additional characterization of thymoquinone’s structure through X-ray diffraction crystallography, they found that the most likely mechanism of electron absorption was via the donation of the radical to the pi bond containing a ring structure and through the reduction of a carbonyl group to a C-OH group, converting the superoxide into O_2_ [60]. Another in vitro study carried out by Gaschler et al. determined that thymoquinone possesses substantial hydroxyl radical and hydrogen peroxide scavenging ability in addition to preventing lipid peroxidation, a process that plays a large role in initiating cell apoptosis [61].

In addition to its direct antioxidant activity, Nrf2 pathway activation has also been reported in several studies with the use of thymoquinone. Velagapudi et al. found that thymoquinone inhibited lipopolysaccharide-induced neuroinflammation through the antioxidative effect of the Nrf2 pathway oxidation in a microglia cell line [62]. When cultured with 2.5 μM, 5 μM, and 10 μM of thymoquinone, a significantly higher amount of Nrf2 protein was localized in the nuclei of the cells, alongside higher levels of Nrf2-related antioxidant enzymes compared to the control. Crucially, the protection against neuroinflammation was reversed when using small interfering RNA to knock down the Nrf2 gene [62]. Similarly, Dong et al. examined thymoquinone’s ability to prevent dopaminergic neurodegeneration through the Nrf2 pathway [63]. They showed that, in vitro, 0.25, 0.5, and 0.75 μM of thymoquinone increased the viability of differentiated neuroblastoma cells when exposed to 1-methyl-4-phenylpyridinium, which is known to induce neurodegeneration [63]. The study also showed increased Nrf2 protein nucleus localization and increased levels of its associated antioxidant enzymes, and when using small interfering RNA to knock down the Nrf2 gene, neurodegeneration would resume [63].

Continuing from their in vitro findings, Dong et al. tested for similar neurodegeneration protection in mice when exposed to the same neurodegenerating compound mentioned above. They found a significant reduction in oxidative stress markers in the mice injected daily with 10 mg/kg of thymoquinone, along with increased Nrf2 protein levels, increased Nrf2 pathway antioxidant enzymes, and protection against neurodegeneration [63]. Once more, they used small interfering RNA to confirm Nrf2 pathway activation as being key to preventing 1-methyl-4-phenylpyridinium damage in the mice [63]. In another study where nephrotoxicity was induced with mercury (II) chloride in rats, thymoquinone protected kidneys from the damage [64]. Sabir et al. found that oral administration of 5, 10, and 15 mg/kg/day of thymoquinone one hour before HgCl_2_ administration prevented nephrotoxicity through the upregulation of the Nrf2 pathway, with 5 mg/kg being the most effective [64]. Increased mRNA levels of Nrf2 protein and increased levels of antioxidant enzymes associated with the Nrf2 pathway were found, and overall biomarkers for oxidative stress were lowered [64]. Lastly, thymoquinone has also effectively warded off the oxidative stress associated with radiation therapy in rats [65]. Doğru et al. injected 50 mg/kg of thymoquinone daily to rats, finding that it was effective in lowering the oxidative stress markers associated with regular gamma radiation exposure compared to control, including oxidative stress index, lipid hydroperoxide, and total oxidant status [65].

Finally, fewer studies have been conducted directly examining the antioxidant effect of thymoquinone and black seed on humans, but the evidence for its capability is promising. In a meta-analysis of five randomized controlled trials conducted by Ardiana et al., it was found that black seed consumption was linked to higher superoxide dismutase levels compared to placebo [66]. Furthermore, Shoaei-Hagh et al. carried out a randomized, double-blind, placebo-controlled trial in hypertensive patients to examine the effects of black seed supplementation and its effect on known cardiovascular risk factors [67]. The patients in the experimental group were given 2.5 mL of black seed oil twice daily, which the investigators calculated as being 33 mg/day of thymoquinone through high-pressure liquid chromatography analysis of their supply of black seed oil [67]. There was a significant reduction in serum malondialdehyde and a significant elevation in glutathione reductase and serum HDL levels in the treatment group, suggesting an antioxidative effect from thymoquinone. There was also a significant reduction in systolic blood pressure, diastolic blood pressure, fasting blood sugar levels, and serum LDL levels over the 8-week experimental period. Notably, the authors emphasize that these outcomes were possibly a result of reducing oxidative stress in the patients in the treatment group [67]. A similar result was found by Ali et al. when combining daily thymoquinone supplementation with metformin, with a significantly higher reduction found in hemoglobin A1C compared to metformin alone in patients with type 2 diabetes over a 90-day period [68].

### 5.2. Conclusions on Black Seed

Overall, there is increasing evidence that the antioxidative effects of black seed and thymoquinone are exhibited through the direct antioxidative potential of thymoquinone, as well as the activation of the Nrf2 pathway. With substantial direct evidence of in vivo Nrf2 activation, it is very likely that a similar activation may occur in humans and would be the subject of further research. There is also preliminary evidence suggesting the utility of direct black seed or thymoquinone supplementation in diseases implicated in oxidative stress. Although the mentioned studies do not have substantially large sample sizes, there is a promising case for its therapeutic use given the potential benefits and lack of side effects. Further studies may be beneficial to ascertain thymoquinone and black seed’s effects on a wider portion of the population.

## 6. Berries and Moringa Oleifera

Berries such as strawberries, blueberries, and many other common berries have been found to have antioxidant effects due to the presence of components such as quercetin, kaempferol, and pterostilbene [69]. Recently, components such as quercetin, kaempferol, and pterostilbene were found to ameliorate oxidative stress through involvement in Nrf2. Nuclear factor Nrf2 is a well-known oxidative stress regulator due to its inhibition of ROS [70]. Through this pathway, the antioxidants quercetin, kaempferol, and pterostilbene, which are common in berries, will be evaluated on how they reduce the oxidative stress through involvement in the Nrf2 pathway.

*Moringa oleifera* (MO), commonly referred to as horseradish tree or drumstick tree, is a tree from the family *Moringacae*, native to Pakistan and north India [71]. Traditionally, leaves, bark, or seeds from MO have been used as a treatment for various ailments due to their high concentration of flavonoids, isothiocyanates, and glucosinolates [72,73,74]. Despite being botanically distinct, berries and MO have multiple overlapping bioactive compounds, including flavanols and phenolic acids [52,70,75].

Among other things, diabetes mellitus (DM) has been linked to impaired mitochondrial function or the stimulation of enzymes such as NADPH oxidase 4 (Nox4) that can contribute to excessive generation of ROS [75,76]. ROS in excess of a cell’s antioxidant capabilities can lead to the damage of lipids, nucleic acids, or proteins, impeding the effects of signaling pathways or increasing the prevalence of cancers [77].

### 6.1. Quercetin

Quercetin is a common flavonoid in berries that is absorbed in the intestine that reduces oxidative stress through involvement with Nrf2 [78]. Quercetin, once absorbed, upregulates the Nrf2 mRNA expressions, cascading into an increase in hemeoxygenase-1(HO-1) levels, which oversee the downregulation of the pro-inflammatory cytokines TNF-α and IL-6 and enzyme-inducible nitric oxide synthase (iNOS), which are common oxidative stress contributors [79]. Shao et al. also reported that quercetin reduced liver and renal oxidative stress through the Nrf2 pathway using Nrf2 knockout mice [80].

MO also contains quercetin, a flavonoid primarily found in the leaves and seeds of MO that lowers Nox4 expression and activates signal pathways that amplify the production of antioxidant compounds, such as the Nrf2 pathway [81,82].

Nrf2 is a protein that, after being released from its repressor receptor, Keap1, translocates to the nucleus and binds to specific DNA sequences called antioxidant response elements (AREs) that encode for antioxidant proteins [83]. By lowering total DNA methyltransferase activity, quercetin decreased Nrf2 sequence methylation, significantly reducing nickel-induced Nrf2 inhibition in the livers of mice. After measuring the density of Nrf2 present in the nucleus, both 40 and 80 mg quantities of quercetin with nickel showed considerably higher concentrations of Nrf2 than the livers of the mice exposed only to nickel [84]. Additionally, when STZ-induced diabetic mice were exposed to 200 mg/kg concentrations of kaempferol, the resulting kidney homogenates had less than half the ROS concentration as the kidney homogenates of STZ-induced diabetic mice that were left unexposed to kaempferol [85]. The structures of both quercetin and kaempferol also give both compounds effective capabilities as ROS scavengers, which can further reduce the damage performed by the overproduction of ROS [86]. This is most notable due to the presence of a catechol group. This allows quercetin, through the donation of hydrogen atoms, to effectively neutralize free radicals, including many ROS [87].

### 6.2. Kaempferol

Kaempferol is a common flavonoid found in berries and MO [88]. Yang et al. showed that kaempferol increased HO-1 through a ROS-dependent Nrf2 pathway using human pulmonary alveolar epithelial cells [89].

Kaempferol may also exhibit some anticancer capabilities. In a study involving various human cancer cell lines, kaempferol showed increasing effectiveness against the survival rates of the cancer cells, even impairing the survival rate of MCF-7 breast cancer cells to 47.89% after exposure to a 100 µg/mL concentration of kaempferol [90]. In another study involving human breast cancer MDA-MB-453 cells, the number of human breast cancer cells in the G1 phase decreased from 85.48% to 51.35% after being treated with kaempferol. The number of cancer cells in G2 increased, however, from 9.27% to 37.5% between the untreated and kaempferol-treated groups [91]. Additionally, when STZ-induced diabetic mice were exposed to 200 mg/kg concentrations of kaempferol, the resulting kidney homogenates had less than half the ROS concentration as the kidney homogenates of STZ-induced diabetic mice that were left unexposed to kaempferol [68]. STZ is a glucose analog that selectively enters pancreatic beta cells via the GLUT 2 transporter. Once inside the cell, STZ acts as an alkylating agent, damaging and fragmenting DNA [92,93]. In patients with DM, hyperglycemia can often result in higher levels of ROS, largely due to protein kinase C and Nox4 activation [94].

### 6.3. Chlorogenic Acid

Chlorogenic acid (CGA), a phenolic acid commonly found in the leaves of MO and some berries, particularly blueberries, may activate AMP-activated protein kinase (AMPK), allowing for an increase in glucose transport into cells [95,96]. When treated with 2 mmol CGA, L6 skeletal muscle cells showed a 14% increase in 2-deoxyglucose uptake over the basal uptake of cells after 1 h of incubation, and a 60% increase over basal uptake after 24 h of incubation. No increase over basal uptake was seen in cells incubated with both CGA- and RNA-silenced AMPK [95]. By lowering blood glucose, oxidative stress may be diminished [97]. CGA also showed some antioxidant capabilities by acting as a ROS scavenger, enhancing Nrf2 translocation into the nucleus, and stimulating other antioxidant enzymes, including catalase and superoxide dismutase [96,98,99].

### 6.4. Combination of Quercetin, Kaempferol, and Pterostilbene

The individual supplements that are used in medication matter. However, drug combinations are also considered as productive methods to increase the effectiveness of the treatment of complex diseases [100]. Individual supplements of quercetin and kaempferol have been experimented with for reducing oxidative stress with the activation of the Nrf2 pathway. Additionally, in a study with rat models, the combination of quercetin and kaempferol and the contribution of polyphenol pterostilbene were investigated for their synergy in increasing the activation of the Nrf2 pathway [69]. Lower dosages of each supplement of quercetin, kaempferol, and pterostilbene were combined for oral gavage. The results indicated that an increase in Nrf2 pathway activity led to protection from oxidants such as hydrogen peroxide [69].

## 7. Holy Basil

Holy basil, or “Tulsi,” has been investigated due to its suspected antioxidative properties and possible treatment of certain cancers [101,102]. Holy basil is edible and contains eugenol, polyphenols, flavonoids, and terpenes within its leaves, which make the plant very widely accessible and used in India [103]. Eugenol is a main component of the antioxidative capabilities of the plant and has a role in inhibiting colon cancer and skin tumors via the Nrf2 pathway [101,102]. Holy basil is also suspected to have a positive impact on DM in reducing oxidative stress [104,105].

### Holy Basil Mechanism of Action

Eugenol has been found to act on the mechanism of action of holy basil by activating the Nrf2 pathway [102]. The activation of the Nrf2 pathway by eugenol contributes to the production of antioxidant and anti-inflammatory molecules, such as superoxide dismutase (SOD) and glutathione peroxidases (GPX), which suppresses ROS production and could be useful in creating a new drug target [106,107].

According to the findings of Ma et al. [106], the transcriptional activity of Nrf2 was significantly increased in both HEK-293 and NIH-3T3 cells that were treated with eugenol compared to the control. Eugenol at 100 µg/mL was found to significantly increase cell viability in the presence of H_2_O_2_ at 50–100 µM. Western blotting showed that the expression levels of the Nrf2 protein were increased by treatment with eugenol in HEK-293 and NIH-3T3 cells. RT-qPCR revealed that eugenol treatment significantly increased mRNA expression of the three target genes downstream of Nrf2 activation (*GCL-M*, *GSTA1*, and *NFE2L2*). The data suggests that eugenol can promote the expression and transcriptional activity of Nrf2, upregulate downstream genes, and increase cell viability in HEK-293 and NIH-3T3 colon cancer cells. Eugenol activates Nrf2 signaling, resulting in the protection of cells from damage from oxidative stress. Eugenol is suspected to be a free radical scavenger by using a hydroxyl group found on its aromatic ring to neutralize ROS [108]. Due to the significant Nrf2 pathway activation and ROS neutralization via eugenol, holy basil could possibly lead to potent antioxidative drugs [106]. Holy basil’s antioxidative capabilities also have implications for use in treatment for DM.

In a sixteen-week study performed by Halim et al., rats were made diabetic via a dose of 60 mg/kg streptozotocin in citrate buffer and left for 1 month to develop, then treated with holy basil. After 16 weeks, the rats were sacrificed [105]. In diabetic rats undergoing treatment with holy basil alone, plasma glucose fell from 232.4 ± 7.66 mg/dL to 85.22 ± 4.59 mg/dL. Without treatment, HbA1c levels rose to more than double those when treated with holy basil alone. There was also a reduction in lipid peroxidases (LPO) compared to the control, with an increase in superoxide dismutase (SOD), catalase (CAT), glutathione peroxidases (GPX), and glutathione-S-transferases (GST) levels found as an effect of treatment. The data showed that aqueous extract of holy basil leaves was a very effective antioxidant. The extract decreased the oxidative stress in plasma (HbA1c) and erythrocytes, and also decreased glucose levels. LPO can cause oxidative damage via hydroxyl radicals that non-specifically attack biomolecules. A radical can initiate a chain oxidation of polyunsaturated phospholipids, which can lead to the dysfunction of a cell membrane [109]. Radicals can also oxidize transition metals found in the body, which can exacerbate the formation of new ROS [109]. The levels of antioxidants such as CAT were increased upon treatment with holy basil, and these are critical in their role in the defense in the cell due to their ability to neutralize ROS and turn them into products such as water and oxygen [105,110]. It is speculated that the malfunction of this defense leads to degenerative diseases such as DM, Alzheimer’s disease, and Parkinson’s disease [110]. Due to the chronic metabolic disorder of DM inducing lipid peroxidation, protein glycation, and impairing ROS neutralization, it is possible that holy basil could be an effective drug to target the associated dysregulation and oxidative stress in DM [105,111].

## 8. Conclusions

Plants and their metabolites have shown great efficacy at creating significant decreases in ROS and increasing the body’s antioxidative capacities by activating Nrf2 pathways and modulating the Th1/Th2 immune axes. Derivatives of plants such as lentinan from shiitake mushroom, saponins and ginsenosides from *Panax ginseng*, curcumin from turmeric, thymoquinone from black seed, and polyphenols and flavonoids found in moringa oleifera and holy basil, all show great results in reducing oxidative stress and enhancing the body’s innate defense mechanisms, as summarized in Table 1. While differences in bioavailability, dosing (Table 2), and long-term efficacy remain areas of ongoing investigation, the consistent pattern emerging from these studies is clear. Many plant compounds possess multifunctional antioxidative capabilities, both directly reducing free radicals and activating the body’s antioxidant defenses. Their potential in future therapeutic efforts is strong and should continue to be researched and developed, as further investigations can help firmly establish that plants and their metabolites may help to cure or alleviate many of the issues associated with today’s lifestyle diseases (Table 3) and nutrient pool.

## Figures and Tables

**Table 1:** Plants and their respective isolated compounds and active pathways.

Plant	Effective Compound	Pathways Effected
Shitake Mushroom	Lentinan	Modulation of Th1/Th2 immune balance, TLR activation, increased APC signaling, improved CMI response
*Panax ginseng*	Ginsenosides (Rg1, Re)	Enhanced Nrf2 activation, decreased oxidative stress, improved glucose metabolism, reduced AGE formation
Turmeric (*Curcuma longa*)	Curcumin	Nrf2 pathway activation, ROS scavenging, increased antioxidant enzyme expression (SOD, catalase, GPx)
Black Seed (*Nigella sativa*)	Thymoquinone	Nrf2 pathway activation, direct ROS scavenging, reduced neuroinflammation and oxidative damage
Berries (e.g., blueberries, strawberries)	Quercetin, Kaempferol, Pterostilbene	Nrf2 pathway activation, antioxidant enzyme upregulation, ROS reduction
Moringa Oleifera	Quercetin, Kaempferol, Nazarin	Downregulation of PKC/Nox4, Nrf2 pathway activation, improved antioxidant capacity
Holy Basil (*Ocimum tenuiflorum*)	Eugenol	Nrf2 activation, increased SOD, GPx, CAT activity, reduced oxidative stress in diabetic and cancer models

**Table 2:** Plant dosage in humans and animals.

Plant/Compound	Dosage	Route	Notes	References
Lentinan (Shiitake Mushroom)	IV: 2–10 mg/week over 30 min; oral: 8 g/day	Intravenous or oral	IV more effective; oral has no side effects but lower efficacy	[9,31]
*Panax ginseng* (Rg1, Re, Rb1, Rd)	Oral: 1 g twice daily; IV: 1–2 mL/session, 1–3x per week	Oral or intravenous	Used in pharmacopuncture; oral better established	[27,28,29]
Curcumin (Turmeric)	Oral: 500 mg curcumin + 5–50 mg piperine/day	Oral	Piperine improves curcumin absorption	[43,44]
Thymoquinone (Black Seed)	Oral: 5–15 mg/kg/day in rats; 33 mg/day in humans	Oral	Derived from black seed oil studies and chromatography	[61,62]
Quercetin, Kaempferol, Pterostilbene (Berries)	Oral: quercetin 40–80 mg/day; kaempferol 200 mg/kg in mice	Oral	Shown to be effective in synergy in animal models	[68,69]
Moringa Oleifera (Quercetin, Kaempferol, Nazarin)	Quercetin 40–80 mg/day in mice; kaempferol 200 mg/kg	Oral	Nrf2 modulation and ROS inhibition observed	[11,75]
Eugenol (Holy Basil)	In vitro: 100 µg/mL; oral: 60 mg/kg/day in rats	In vitro and oral (rat model)	Antioxidant and antidiabetic effects in animal studies	[90,91]

**Table 3:** Morbidity and/or diseases that can be affected by plant derived compounds.

Morbidity/Disease	Effective Compound	Physiological Response	References
Diabetes	Red ginseng (*Panax ginseng*) metabolites Rg1 and Re	Lowered blood glucose levels, reduced AGE formation, enhanced antioxidant enzymes (e.g., increased glutathione), improved kidney function, and reduced oxidative stress	[24,28]
Cancer	Lentinan (from shiitake mushroom)	Modulation of Th1/Th2 ratio, increased cytotoxic T-cell (CD8+) and NK cell activity, promotion of apoptosis in cancer cells, reduction in tumor angiogenesis and ROS	[2,3,9]
Obesity	Quercetin, kaempferol, pterostilbene (from berries)	Enhanced Nrf2 activation leading to increased antioxidant enzyme activity, reduced oxidative stress and inflammation associated with metabolic dysfunction	[69,70]
Cardiovascular disease	Curcumin (from turmeric) + piperine	Improved antioxidant capacity, reduced carotid intima–media thickness, enhanced Nrf2 pathway activation, reduced oxidative stress markers	[52,53]
Hypertension	Thymoquinone (from black seed)	Reduced blood pressure, decreased oxidative stress, improved lipid profiles, enhanced antioxidant enzyme activity through Nrf2 pathway	[66,67]

## Abbreviations

The following abbreviations are used in this manuscript:

ROS	Reactive oxygen species
STZ	Streptozotocin
APC	Antigen-presenting cells
IFN-γ	Interferon gamma
NSCLC	Non-small-cell lung cancer
Nrf2	Nuclear factor E2-related factor 2
CMI	Cell-mediated immune response
MPE	Malignant pleural effusion
IBD	Irritable bowel disease
DSS	Dextran sodium sulfate
LDL	Low-density lipoprotein
HDL	High-density lipoprotein
PKC	Protein kinase C
SOD	Superoxide dismutase
GPX	Glutathione peroxidase
HbA1c	Glycated hemoglobin
iNOS	Inducible nitric oxide synthase
HO-1	Heme oxygenase 1
Keap1	Kelch-like ECH-associated protein 1
PT	Prothrombin time
INR	International normalized ratio
AGE	Advanced glycation end-products

## References

[1] Cooper G.M. (2000). The development and causes of cancer. The Cell: A Molecular Approach.

[2] Shang Q., Yu X., Sun Q., Li H., Sun C., Liu L. (2024). Polysaccharides regulate th1/th2 balance: A new strategy for tumor immunotherapy. Biomed. Pharmacother..

[3] Xiao Y., Huang Y., Jiang J., Chen Y., Wei C. (2023). Identification of the prognostic value of th1/th2 ratio and a novel prognostic signature in basal-like breast cancer. Hereditas.

[4] Geindreau M., Bruchard M., Vegran F. (2022). Role of cytokines and chemokines in angiogenesis in a tumor context. Cancers.

[5] Liu W., Gong X., Luo J., Jiang L., Lu W., Pan C., Yao W., Gao X., Tian H. (2021). A purified acidic polysaccharide from sarcandra glabra as vaccine adjuvant to enhance anti-tumor effect of cancer vaccine. Carbohydr. Polym..

[6] Zhou G., Liu H., Yuan Y., Wang Q., Wang L., Wu J. (2024). Lentinan progress in inflammatory diseases and tumor diseases. Eur. J. Med. Res..

[7] Deng S., Zhang G., Kuai J., Fan P., Wang X., Zhou P., Yang D., Zheng X., Liu X., Wu Q. (2018). Lentinan inhibits tumor angiogenesis via interferon gamma and in a t cell independent manner. J. Exp. Clin. Cancer Res..

[8] Song Z., Luo W., Zheng H., Zeng Y., Wang J., Chen T. (2021). Translational nanotherapeutics reprograms immune microenvironment in malignant pleural effusion of lung adenocarcinoma. Adv. Healthc. Mater..

[9] Wang X.E., Wang Y.H., Zhou Q., Peng M., Zhang J., Chen M., Ma L.J., Xie G.M. (2020). Immunomodulatory effect of lentinan on aberrant t subsets and cytokines profile in non-small cell lung cancer patients. Pathol. Oncol. Res..

[10] Liu Y., Zhao J., Zhao Y., Zong S., Tian Y., Chen S., Li M., Liu H., Zhang Q., Jing X. (2019). Therapeutic effects of lentinan on inflammatory bowel disease and colitis-associated cancer. J. Cell. Mol. Med..

[11] Santana P.T., Rosas S.L.B., Ribeiro B.E., Marinho Y., de Souza H.S.P. (2022). Dysbiosis in inflammatory bowel disease: Pathogenic role and potential therapeutic targets. Int. J. Mol. Sci..

[12] Rizzatti G., Lopetuso L.R., Gibiino G., Binda C., Gasbarrini A. (2017). Proteobacteria: A common factor in human diseases. Biomed. Res. Int..

[13] Yang X., Zheng M., Zhou M., Zhou L., Ge X., Pang N., Li H., Li X., Li M., Zhang J. (2021). Lentinan supplementation protects the gut-liver axis and prevents steatohepatitis: The role of gut microbiota involved. Front. Nutr..

[14] Chong W.P., Mattapallil M.J., Raychaudhuri K., Bing S.J., Wu S., Zhong Y., Wang W., Chen Z., Silver P.B., Jittayasothorn Y. (2020). The cytokine il-17a limits th17 pathogenicity via a negative feedback loop driven by autocrine induction of il-24. Immunity.

[15] Ross D., Siegel D. (2021). The diverse functionality of nqo1 and its roles in redox control. Redox Biol..

[16] Yang Y., Song S., Nie Y., Chen R., Chen P. (2022). Lentinan alleviates arsenic-induced hepatotoxicity in mice via downregulation of ox40/il-17a and activation of nrf2 signaling. BMC Pharmacol. Toxicol..

[17] Gordon M., Bihari B., Goosby E., Gorter R., Greco M., Guralnik M., Mimura T., Rudinicki V., Wong R., Kaneko Y. (1998). A placebo-controlled trial of the immune modulator, lentinan, in hiv-positive patients: A phase i/ii trial. J. Med..

[18] deVere White R.W., Hackman R.M., Soares S.E., Beckett L.A., Sun B. (2002). Effects of a mushroom mycelium extract on the treatment of prostate cancer. Urology.

[19] Gabriel M.F., Gonzalez-Delgado P., Postigo I., Fernandez J., Soriano V., Cueva B., Martinez J. (2015). From respiratory sensitization to food allergy: Anaphylactic reaction after ingestion of mushrooms (agaricus bisporus). Med. Mycol. Case Rep..

[20] Lin Y., Wei Y., Hu X., Wu M., Yao J., Ying X., Fu X., Ding M., Qiao L. (2019). Evaluation of lentinan effects on cytochrome p450 activity in rats by a cocktail method. Iran. J. Basic. Med. Sci..

[21] Clark N.P., Hoang K., Delate T., Horn J.R., Witt D.M. (2018). Warfarin interaction with hepatic cytochrome p-450 enzyme-inducing anticonvulsants. Clin. Appl. Thromb. Hemost..

[22] Gkogkolou P., Bohm M. (2012). Advanced glycation end products: Key players in skin aging?. Dermatoendocrinol.

[23] Baynes J.W. (1991). Role of oxidative stress in development of complications in diabetes. Diabetes.

[24] Hsu R.K., Hsu C.Y. (2011). Proteinuria and reduced glomerular filtration rate as risk factors for acute kidney injury. Curr. Opin. Nephrol. Hypertens..

[25] Yun T.K. (2001). Brief introduction of panax ginseng c.A. Meyer. J. Korean Med. Sci..

[26] Park J.H., Kim J.M., Han S.B., Kim N.Y., Surh Y.J., Lee S.K., Kim N.D., Park M.K. (1975). A new processed ginseng with fortified activity. Proceedings of the Ginseng Society Conference.

[27] Chen H., Yin J., Deng Y., Yang M., Xu L., Teng F., Li D., Cheng Y., Liu S., Wang D. (2012). The protective effects of ginsenoside rg1 against hypertension target-organ damage in spontaneously hypertensive rats. BMC Complement. Altern. Med..

[28] Cho W.C., Chung W.S., Lee S.K., Leung A.W., Cheng C.H., Yue K.K. (2006). Ginsenoside re of panax ginseng possesses significant antioxidant and antihyperlipidemic efficacies in streptozotocin-induced diabetic rats. Eur. J. Pharmacol..

[29] Wang G., Lei C., Tian Y., Wang Y., Zhang L., Zhang R. (2019). Rb1, the primary active ingredient in panax ginseng c.A. Meyer, exerts antidepressant-like effects via the bdnf-trkb-creb pathway. Front. Pharmacol..

[30] Zhang H.A., Wang M., Zhou J., Yao Q.Y., Ma J.M., Jiang C.L. (2010). Protective effect of ginsenoside against acute renal failure and expression of tyrosine hydroxylase in the locus coeruleus. Physiol. Res..

[31] Sun Q., Meng Q.T., Jiang Y., Xia Z.Y. (2012). Ginsenoside rb1 attenuates intestinal ischemia reperfusion induced renal injury by activating nrf2/are pathway. Molecules.

[32] Lee M.Y., Singh D., Kim S.H., Lee S.J., Lee C.H. (2016). Ultrahigh pressure processing produces alterations in the metabolite profiles of panax ginseng. Molecules.

[33] Kang K.S., Ham J., Kim Y.J., Park J.H., Cho E.J., Yamabe N. (2013). Heat-processed panax ginseng and diabetic renal damage: Active components and action mechanism. J. Ginseng Res..

[34] Yokozawa T., Owada S. (1999). Effect of ginsenoside-rd in cephaloridine-induced renal disorder. Nephron.

[35] Yokozawa T., Liu Z.W., Dong E. (1998). A study of ginsenoside-rd in a renal ischemia-reperfusion model. Nephron.

[36] Lee I.S., Kang K.S., Kim S.Y. (2019). Panax ginseng pharmacopuncture: Current status of the research and future challenges. Biomolecules.

[37] Yuan C.S., Wei G., Dey L., Karrison T., Nahlik L., Maleckar S., Kasza K., Ang-Lee M., Moss J. (2004). Brief communication: American ginseng reduces warfarin’s effect in healthy patients: A randomized, controlled trial. Ann. Intern. Med..

[38] Malati C.Y., Robertson S.M., Hunt J.D., Chairez C., Alfaro R.M., Kovacs J.A., Penzak S.R. (2012). Influence of panax ginseng on cytochrome p450 (cyp)3a and p-glycoprotein (p-gp) activity in healthy participants. J. Clin. Pharmacol..

[39] Paik D.J., Lee C.H. (2015). Review of cases of patient risk associated with ginseng abuse and misuse. J. Ginseng Res..

[40] Qin S., Huang L., Gong J., Shen S., Huang J., Ren H., Hu H. (2017). Efficacy and safety of turmeric and curcumin in lowering blood lipid levels in patients with cardiovascular risk factors: A meta-analysis of randomized controlled trials. Nutr. J..

[41] Kocaadam B., Sanlier N. (2017). Curcumin, an active component of turmeric (*Curcuma longa*), and its effects on health. Crit. Rev. Food Sci. Nutr..

[42] Hajam Y.A., Rani R., Ganie S.Y., Sheikh T.A., Javaid D., Qadri S.S., Pramodh S., Alsulimani A., Alkhanani M.F., Harakeh S. (2022). Oxidative stress in human pathology and aging: Molecular mechanisms and perspectives. Cells.

[43] Ak T., Gulcin I. (2008). Antioxidant and radical scavenging properties of curcumin. Chem. Biol. Interact..

[44] Keramat M., Golmakani M.T. (2024). Antioxidant potency and inhibitory mechanism of curcumin and its derivatives in oleogel and emulgel produced by linseed oil. Food Chem..

[45] Baird L., Yamamoto M. (2020). The molecular mechanisms regulating the keap1-nrf2 pathway. Mol. Cell Biol..

[46] Zia A., Farkhondeh T., Pourbagher-Shahri A.M., Samarghandian S. (2021). The role of curcumin in aging and senescence: Molecular mechanisms. Biomed. Pharmacother..

[47] Lin X., Bai D., Wei Z., Zhang Y., Huang Y., Deng H., Huang X. (2019). Curcumin attenuates oxidative stress in raw264.7 cells by increasing the activity of antioxidant enzymes and activating the nrf2-keap1 pathway. PLoS ONE.

[48] Liu C., Rokavec M., Huang Z., Hermeking H. (2023). Curcumin activates a ros/keap1/nrf2/mir-34a/b/c cascade to suppress colorectal cancer metastasis. Cell Death Differ..

[49] Al-Rubaei Z.M., Mohammad T.U., Ali L.K. (2014). Effects of local curcumin on oxidative stress and total antioxidant capacity in vivo study. Pak. J. Biol. Sci..

[50] Pullarkat V., Meng Z., Tahara S.M., Johnson C.S., Kalra V.K. (2014). Proteasome inhibition induces both antioxidant and hb f responses in sickle cell disease via the nrf2 pathway. Hemoglobin.

[51] Jakubczyk K., Druzga A., Katarzyna J., Skonieczna-Zydecka K. (2020). Antioxidant potential of curcumin-a meta-analysis of randomized clinical trials. Antioxidants.

[52] Boshagh K., Khorvash F., Sahebkar A., Majeed M., Bahreini N., Askari G., Bagherniya M. (2023). The effects of curcumin-piperine supplementation on inflammatory, oxidative stress and metabolic indices in patients with ischemic stroke in the rehabilitation phase: A randomized controlled trial. Nutr. J..

[53] Sharifi S., Bagherniya M., Khoram Z., Ebrahimi Varzaneh A., Atkin S.L., Jamialahmadi T., Sahebkar A., Askari G. (2023). Efficacy of curcumin plus piperine co-supplementation in moderate-to-high hepatic steatosis: A double-blind, randomized, placebo-controlled clinical trial. Phytother. Res..

[54] Li G., Fang S., Shao X., Li Y., Tong Q., Kong B., Chen L., Wang Y., Yang J., Yu H. (2021). Curcumin reverses nnmt-induced 5-fluorouracil resistance via increasing ros and cell cycle arrest in colorectal cancer cells. Biomolecules.

[55] Xu C., Wang M., Zandieh Doulabi B., Sun Y., Liu Y. (2023). Paradox: Curcumin, a natural antioxidant, suppresses osteosarcoma cells via excessive reactive oxygen species. Int. J. Mol. Sci..

[56] El Assar M., Alvarez-Bustos A., Sosa P., Angulo J., Rodriguez-Manas L. (2022). Effect of physical activity/exercise on oxidative stress and inflammation in muscle and vascular aging. Int. J. Mol. Sci..

[57] Vollono L., Falconi M., Gaziano R., Iacovelli F., Dika E., Terracciano C., Bianchi L., Campione E. (2019). Potential of curcumin in skin disorders. Nutrients.

[58] Kooti W., Hasanzadeh-Noohi Z., Sharafi-Ahvazi N., Asadi-Samani M., Ashtary-Larky D. (2016). Phytochemistry, pharmacology, and therapeutic uses of black seed (*Nigella sativa*). Chin. J. Nat. Med..

[59] Hannan M.A., Rahman M.A., Sohag A.A.M., Uddin M.J., Dash R., Sikder M.H., Rahman M.S., Timalsina B., Munni Y.A., Sarker P.P. (2021). Black cumin (*Nigella sativa* L.): A comprehensive review on phytochemistry, health benefits, molecular pharmacology, and safety. Nutrients.

[60] Sakib R., Caruso F., Aktar S., Belli S., Kaur S., Hernandez M., Rossi M. (2023). Antioxidant properties of thymoquinone, thymohydroquinone and black cumin (*Nigella sativa* L.) seed oil: Scavenging of superoxide radical studied using cyclic voltammetry, dft and single crystal x-ray diffraction. Antioxidants.

[61] Gaschler M.M., Stockwell B.R. (2017). Lipid peroxidation in cell death. Biochem. Biophys. Res. Commun..

[62] Velagapudi R., Kumar A., Bhatia H.S., El-Bakoush A., Lepiarz I., Fiebich B.L., Olajide O.A. (2017). Inhibition of neuroinflammation by thymoquinone requires activation of nrf2/are signalling. Int. Immunopharmacol..

[63] Dong J., Zhang X., Wang S., Xu C., Gao M., Liu S., Li X., Cheng N., Han Y., Wang X. (2020). Thymoquinone prevents dopaminergic neurodegeneration by attenuating oxidative stress via the nrf2/are pathway. Front. Pharmacol..

[64] Sabir S., Saleem U., Akash M.S.H., Qasim M., Chauhdary Z. (2022). Thymoquinone induces nrf2 mediated adaptive homeostasis: Implication for mercuric chloride-induced nephrotoxicity. ACS Omega.

[65] Dogru S., Taysi S., Yucel A. (2024). Effects of thymoquinone in the lungs of rats against radiation-induced oxidative stress. Eur. Rev. Med. Pharmacol. Sci..

[66] Ardiana M., Pikir B.S., Santoso A., Hermawan H.O., Al-Farabi M.J. (2020). Effect of *Nigella sativa* supplementation on oxidative stress and antioxidant parameters: A meta-analysis of randomized controlled trials. Sci. World J..

[67] Shoaei-Hagh P., Kamelan Kafi F., Najafi S., Zamanzadeh M., Heidari Bakavoli A., Ramezani J., Soltanian S., Asili J., Hosseinzadeh H., Eslami S. (2021). A randomized, double-blind, placebo-controlled, clinical trial to evaluate the benefits of *Nigella sativa* seeds oil in reducing cardiovascular risks in hypertensive patients. Phytother. Res..

[68] Ali S.M., Chen P., Sheikh S., Ahmad A., Ahmad M., Paithankar M., Desai B., Patel P., Khan M., Chaturvedi A. (2021). Thymoquinone with metformin decreases fasting, post prandial glucose, and hba1c in type 2 diabetic patients. Drug Res..

[69] Saw C.L., Guo Y., Yang A.Y., Paredes-Gonzalez X., Ramirez C., Pung D., Kong A.N. (2014). The berry constituents quercetin, kaempferol, and pterostilbene synergistically attenuate reactive oxygen species: Involvement of the nrf2-are signaling pathway. Food Chem. Toxicol..

[70] Zhang L., Xu L.Y., Tang F., Liu D., Zhao X.L., Zhang J.N., Xia J., Wu J.J., Yang Y., Peng C. (2024). New perspectives on the therapeutic potential of quercetin in non-communicable diseases: Targeting nrf2 to counteract oxidative stress and inflammation. J. Pharm. Anal..

[71] Patil S.V., Mohite B.V., Marathe K.R., Salunkhe N.S., Marathe V., Patil V.S. (2022). Moringa tree, gift of nature: A review on nutritional and industrial potential. Curr. Pharmacol. Rep..

[72] Fahey J.W., Wade K.L., Stephenson K.K., Shi Y., Liu H., Panjwani A.A., Warrick C.R., Olson M.E. (2019). A strategy to deliver precise oral doses of the glucosinolates or isothiocyanates from moringa oleifera leaves for use in clinical studies. Nutrients.

[73] Jaja-Chimedza A., Graf B.L., Simmler C., Kim Y., Kuhn P., Pauli G.F., Raskin I. (2017). Biochemical characterization and anti-inflammatory properties of an isothiocyanate-enriched moringa (*Moringa oleifera*) seed extract. PLoS ONE.

[74] Stohs S.J., Hartman M.J. (2015). Review of the safety and efficacy of moringa oleifera. Phytother. Res..

[75] Pearce B., Pearce K. (2024). Mitochondrial dysfunction and diabetes in south africa: A review. Endocr. Metab. Sci..

[76] Wang F., Bao Y., Shen X., Zengin G., Lyu Y., Xiao J., Weng Z. (2021). Niazirin from moringa oleifera lam. Attenuates high glucose-induced oxidative stress through pkczeta/nox4 pathway. Phytomedicine.

[77] Burgos-Moron E., Abad-Jimenez Z., Maranon A.M., Iannantuoni F., Escribano-Lopez I., Lopez-Domenech S., Salom C., Jover A., Mora V., Roldan I. (2019). Relationship between oxidative stress, er stress, and inflammation in type 2 diabetes: The battle continues. J. Clin. Med..

[78] Rein M.J., Renouf M., Cruz-Hernandez C., Actis-Goretta L., Thakkar S.K., da Silva Pinto M. (2013). Bioavailability of bioactive food compounds: A challenging journey to bioefficacy. Br. J. Clin. Pharmacol..

[79] Aghababaei F., Hadidi M. (2023). Recent advances in potential health benefits of quercetin. Pharmaceuticals.

[80] Shao Y., Yu H., Yang Y., Li M., Hang L., Xu X. (2019). A solid dispersion of quercetin shows enhanced nrf2 activation and protective effects against oxidative injury in a mouse model of dry age-related macular degeneration. Oxid. Med. Cell. Longev..

[81] Alrumaihi F., Almatroodi S.A., Alharbi H.O.A., Alwanian W.M., Alharbi F.A., Almatroudi A., Rahmani A.H. (2024). Pharmacological potential of kaempferol, a flavonoid in the management of pathogenesis via modulation of inflammation and other biological activities. Molecules.

[82] Sultana B., Anwar F. (2008). Flavonols (kaempeferol, quercetin, myricetin) contents of selected fruits, vegetables and medicinal plants. Food Chem..

[83] Hussain Y., Khan H., Alsharif K.F., Hayat Khan A., Aschner M., Saso L. (2022). The therapeutic potential of kaemferol and other naturally occurring polyphenols might be modulated by nrf2-are signaling pathway: Current status and future direction. Molecules.

[84] Liu C.M., Ma J.Q., Xie W.R., Liu S.S., Feng Z.J., Zheng G.H., Wang A.M. (2015). Quercetin protects mouse liver against nickel-induced DNA methylation and inflammation associated with the nrf2/ho-1 and p38/stat1/nf-kappab pathway. Food Chem. Toxicol..

[85] Alshehri A.S. (2023). Kaempferol attenuates diabetic nephropathy in streptozotocin-induced diabetic rats by a hypoglycaemic effect and concomitant activation of the nrf-2/ho-1/antioxidants axis. Arch. Physiol. Biochem..

[86] Speisky H., Arias-Sante M.F., Fuentes J. (2023). Oxidation of quercetin and kaempferol markedly amplifies their antioxidant, cytoprotective, and anti-inflammatory properties. Antioxidants.

[87] Veiko A.G., Lapshina E.A., Zavodnik I.B. (2021). Comparative analysis of molecular properties and reactions with oxidants for quercetin, catechin, and naringenin. Mol. Cell Biochem..

[88] Chen A.Y., Chen Y.C. (2013). A review of the dietary flavonoid, kaempferol on human health and cancer chemoprevention. Food Chem..

[89] Yang C.C., Hsiao L.D., Wang C.Y., Lin W.N., Shih Y.F., Chen Y.W., Cho R.L., Tseng H.C., Yang C.M. (2022). Ho-1 upregulation by kaempferol via ros-dependent nrf2-are cascade attenuates lipopolysaccharide-mediated intercellular cell adhesion molecule-1 expression in human pulmonary alveolar epithelial cells. Antioxidants.

[90] Liao W., Chen L., Ma X., Jiao R., Li X., Wang Y. (2016). Protective effects of kaempferol against reactive oxygen species-induced hemolysis and its antiproliferative activity on human cancer cells. Eur. J. Med. Chem..

[91] Wang X., Yang Y., An Y., Fang G. (2019). The mechanism of anticancer action and potential clinical use of kaempferol in the treatment of breast cancer. Biomed. Pharmacother..

[92] Marino F., Salerno N., Scalise M., Salerno L., Torella A., Molinaro C., Chiefalo A., Filardo A., Siracusa C., Panuccio G. (2023). Streptozotocin-induced type 1 and 2 diabetes mellitus mouse models show different functional, cellular and molecular patterns of diabetic cardiomyopathy. Int. J. Mol. Sci..

[93] Furman B.L. (2015). Streptozotocin-induced diabetic models in mice and rats. Curr. Protoc. Pharmacol..

[94] Volpe C.M.O., Villar-Delfino P.H., Dos Anjos P.M.F., Nogueira-Machado J.A. (2018). Cellular death, reactive oxygen species (ros) and diabetic complications. Cell Death Dis..

[95] Ong K.W., Hsu A., Tan B.K. (2012). Chlorogenic acid stimulates glucose transport in skeletal muscle via ampk activation: A contributor to the beneficial effects of coffee on diabetes. PLoS ONE.

[96] Bender O., Atalay A., Preedy V.R., Patel V.B. (2021). Chapter 28—Polyphenol chlorogenic acid, antioxidant profile, and breast cancer. Cancer: Oxidative Stress and Dietary Antioxidants.

[97] Tomiyama H., Kushiro T., Okazaki R., Yoshida H., Doba N., Yamashina A. (2003). Influences of increased oxidative stress on endothelial function, platelets function, and fibrinolysis in hypertension associated with glucose intolerance. Hypertens. Res..

[98] Wang D., Hou J., Wan J., Yang Y., Liu S., Li X., Li W., Dai X., Zhou P., Liu W. (2021). Dietary chlorogenic acid ameliorates oxidative stress and improves endothelial function in diabetic mice via nrf2 activation. J. Int. Med. Res..

[99] Zhou Y., Zhou L., Ruan Z., Mi S., Jiang M., Li X., Wu X., Deng Z., Yin Y. (2016). Chlorogenic acid ameliorates intestinal mitochondrial injury by increasing antioxidant effects and activity of respiratory complexes. Biosci. Biotechnol. Biochem..

[100] Pearson R.A., Wicha S.G., Okour M. (2023). Drug combination modeling: Methods and applications in drug development. J. Clin. Pharmacol..

[101] Cohen M.M. (2014). Tulsi—Ocimum sanctum: A herb for all reasons. J. Ayurveda Integr. Med..

[102] Kumar S., Sarkar B. (2023). In silico approach to assessing the polyphenols from krishna tulsi (*Ocimum tenuiflorum* L.) as a keap1/nrf2 receptor for the treatment of inflammatory bowel disease. Med. Sci. Forum.

[103] Ponnusam Y., Louis T., Madhavachandran V., Kumar S., Thoprani N., Hamblin M.R., Lakshmanan M. (2015). Antioxidant activity of the ancient herb, holy basil in ccl4-induced liver injury in rats. Ayurvedic.

[104] De Rango P., Estrera A.L., Miller C., Lee T.Y., Keyhani K., Abdullah S., Safi H. (2011). Operative outcomes using a side-branched thoracoabdominal aortic graft (stag) for thoraco-abdominal aortic repair. Eur. J. Vasc. Endovasc. Surg..

[105] Halim E.M., Mukhopadhyay A.K. (2006). Effect ofocimum sanctum (tulsi) and vitamin e on biochemical parameters and retinopathy in streptozotocin induced diabetic rats. Indian. J. Clin. Biochem..

[106] Ma Q. (2013). Role of nrf2 in oxidative stress and toxicity. Annu. Rev. Pharmacol. Toxicol..

[107] Wang D., Zhang T., Ye H., Hao H., Zhang H., Zhao C. (2020). In vitro probiotic screening and evaluation of space-induced mutant lactobacillus plantarum. Food Sci. Nutr..

[108] Sohilait H.J., Kainama H. (2019). Free radical scavenging activity of essential oil of eugenia caryophylata from amboina island and derivatives of eugenol. Open Chem..

[109] Ayala A., Munoz M.F., Arguelles S. (2014). Lipid peroxidation: Production, metabolism, and signaling mechanisms of malondialdehyde and 4-hydroxy-2-nonenal. Oxid. Med. Cell. Longev..

[110] Kepka S., Lemaitre L., Marx T., Bilbault P., Desmettre T. (2019). A common gesture with a rare but potentially severe complication: Re-expansion pulmonary edema following chest tube drainage. Respir. Med. Case Rep..

[111] Jilani A., Hussain S.Z., Melaibari A.A., Abu-Hamdeh N.H. (2022). Development and mechanistic studies of ternary nanocomposites for hydrogen production from water splitting to yield sustainable/green energy and environmental remediation. Polymers.

